# The Relationships between Total Protein Intake, Protein Sources, Physical Activity, and Lean Mass in a Representative Sample of the US Adults

**DOI:** 10.3390/nu12103151

**Published:** 2020-10-15

**Authors:** Furong Xu, Jacob E. Earp, Maya Vadiveloo, Alessandra Adami, Matthew J. Delmonico, Ingrid E. Lofgren, Mary L. Greaney

**Affiliations:** 1 Department of Kinesiology, University of Rhode Island, Independence Square II, Kingston, RI 02881, USA; aadami@uri.edu (A.A.); delmonico@uri.edu (M.J.D.); 2 Department of Kinesiology, University of Connecticut, Gampel Pavilion, Storrs, CT 06269, USA; jacob.earp@uconn.edu; 3 Department of Nutrition and Food Sciences, University of Rhode Island, Fogarty Hall, Kingston, RI 02881, USA; maya_vadiveloo@uri.edu (M.V.); ingrid_lofgren@uri.edu (I.E.L.); 4 Department of Health Studies, University of Rhode Island, Independence Square II, Kingston, RI 02881, USA; mgreaney@uri.edu

**Keywords:** lean mass, lean mass index, appendicular lean mass, physical activity, total protein intake, protein sources

## Abstract

Background: Although dietary protein and physical activity play essential roles in developing and preserving lean mass, studies exploring these relationships are inconsistent, and large-scale studies on sources of protein and lean mass are lacking. Accordingly, the present study examined the relationship between total protein intake, protein sources, physical activity, and lean mass in a representative sample of US adults. Methods: This cross-sectional study analyzed data from 2011–2016 US National Health and Nutrition Examination Survey and corresponding Food Patterns Equivalents Database (*n* = 7547). Multiple linear regression models were performed to examine the sex-specific associations between total protein intake, protein sources (Dairy, Total Protein Foods, Seafood, and Plant Proteins), physical activity, and lean mass adjusting for demographics, weight status, and total daily energy intake. Results: Total protein intake was inversely related to lean mass in females only (Lean mass index: β= −0.84, 95%CI: −1.06–−0.62; Appendicular lean mass index: β= −0.35, 95%CI: −0.48–−0.22). However, protein sources and physical activity was positively associated with lean mass in males and/or females (*p* < 0.05). Conclusion. Study results suggest that consuming more protein daily had a detrimental influence on lean mass in females whereas eating high-quality sources of proteins and being physically active are important for lean mass for men and women. However, the importance of specific protein sources appears to differ by sex and warrants further investigation.

## 1. Introduction

Lean mass is important for optimal health given its relationship with quality of life [1], the risk of chronic diseases (e.g., cardiovascular disease) [2,3], and mortality [2,4]. Consuming the recommended daily protein intake and being physically active are crucial to lean mass development and preservation [5,6,7]. However, studies, especially large-scale studies examining the relationships between protein intake and/or physical activity and lean mass in adults are scarce, specifically studies using objective measures of lean mass such as dual-energy X-ray absorptiometry (DXA) [8,9,10,11], body composition analyzer [12], and bioelectrical impedance analysis [13]. Also, the findings about the relationships between total protein intake and/or physical activity and lean mass are inconsistent. Supportive results have identified positive relationships between protein consumption frequency [8], or total protein intake [9,12] and lean mass. Other studies have indicated that total protein intake was inversely related [10] or unrelated [13] to lean mass index (LMI). Similarly, research findings examining the relationship between physical activity and lean mass are inconsistent [8,11]. One study identified a positive association between physical activity and lean mass [8], whereas another study found that physical activity was positively related to lean mass in females only [11]. Given inconsistent findings and use of varied measurements, there is a need for more research investigating the relationship of total protein intake and physical activity with lean mass in a representative adult population, especially lean mass indices derived using DXA outputs, one of the most accurate lean mass measures [14].

Although several of the aforementioned large-scale studies have examined the relationship between total protein intake and lean mass [9,10,12,13], no similar large-scale studies have examined the relationship between protein sources and lean mass. Total protein consumption does not take into account the sources of the protein consumed, which may play an important role in the development and maintenance of lean mass as protein sources differ in their amino acid composition which has been shown to affect muscle growth [15,16]. This may be of particular interest as dietary avoidance of specific protein sources (e.g., meats or dairy) are common due to dietary intolerances, ethical concerns, or dietary preferences [15,17,18]. The 2015–2020 Dietary Guidelines for Americans focuses on eating a variety of protein sources rather than a food or a food group for optimal health while considering individuals’ overall dietary pattern [19]. Thus, there is a necessity to examine the relationship between protein sources which was measured based on the 2015–2020 Dietary Guidelines for Americans and lean mass. Accordingly, the primary aim of the present study was to examine the relationships between total protein intake, sources of protein, physical activity, and lean mass indices in a representative sample of US adults. Moreover, since both dietary protein and physical activity are important for lean mass [6,8], the secondary study aim was to explore the association between integrated physical activity and protein intake and lean mass.

## 2. Methods

This cross-sectional study used three cycles of data (2011–2012, 2013–2014, 2015–2016) from the National Health and Nutrition Examination Survey (NHANES) and corresponding Food Patterns Equivalents Database (FPED) [20,21]. In total, 7547 adults (aged 20 to 59 years) out of 29,902 respondents from 2011–2016 NHANES and corresponding FPED data cycles were included in the study sample using following inclusion criteria: (1) aged 20 years or older, and (2) had completed data for physical activity, two 24-h dietary recalls, and DXA (DXA data only available up to ages 59 years for data cycles used). This study was approved by the University of Rhode Island’s Institutional Review Board (IRB#1620717–1) in the exempt review category according to federal regulations 45 CFR 46.

### 2.1. Total Protein Intake and Protein Sources

Two aspects of protein intake were examined in the present study. The first aspect was quantity of total protein intake, grams per kilogram of body weight per day (g/kg/d), was calculated by averaging daily protein intake estimated from two 24-h dietary recalls and dividing by body weight (kg) [20]. Then, according to the recommended minimum daily protein intake of 0.8 g/kg/d for adults, total protein intake was dichotomized as met/not met the daily protein intake recommendation [22].

The second aspect of protein intake that was examined was protein sources. Protein intake by sources was measured via the HEI-2015. The HEI-2015 and its 13 components are calculated using the simple HEI scoring algorithm method using data from two 24-h dietary recalls [20,23,24]. Each of the 13 components of the HEI-2015 represents an aspect of diet quality and three of components are protein-related: Dairy (0–10), Total Protein Foods (0–5), and Seafood and Plant Proteins (0–5) [23]. A higher component score indicates better adherence to the 2015–2020 dietary guidelines for that particular aspect of diet quality being assessed. Since no cut points are available in the existing literature, intake was classified as being good or fair/poor based on score distributions: (1) Dairy [good (3rd tertile scores ranged from 6.51 to 10), fair/poor (1st and 2nd tertiles, scores < 6.51)], (2) Total Protein Foods [good (scored 5, 66%), fair/poor (scored < 5, 34%)], (3) Seafood and Plant Proteins [good (scored 5, 60%), fair/poor (scored < 5, 40%)].

### 2.2. Physical Activity

Respondents’ physical activity time (min/week) was collected via the Global Physical Activity Questionnaire [20] in which physical activity encompassed three domains: work, transport, and recreation [25]. Physical activity data were cleaned and analyzed following World Health Organization’s Global Physical Activity Questionnaire analysis guide [25]. Then, respondents estimated physical activity time was dichotomized based on whether they met the current physical activity recommendation (yes vs. no) of at least 150 min/week moderate intensity or 75 min/week vigorous intensity physical activity or an equivalent combination of both [26].

### 2.3. Lean Mass Indices

The lean mass measures, LMI and appendicular lean mass index (ALMI), used in the present study were derived from DXA (Hologic, Inc., Bedford, MA, USA) outputs of total and regional lean mass excluding bone mineral content (BMC). Specifically, LMI (kg/m^2^) was calculated by using total lean mass excluding BMC divided by height in meters squared; ALMI (kg/m^2^) is a measure of lean mass in arms and legs excluding BMC divided by height in meters squared [13]. Then LMI and ALMI for total, males, females were classified into different levels based on tertiles: low (1st tertile), middle (2nd tertile), and high (3rd tertile).

### 2.4. Physical Activity and Protein Combinations

Respondents were categorized into integrated groups based on whether they met the physical activity recommendation and their protein intake (daily protein intake recommendation, protein sources classifications). The following groups were created: (1) met physical activity recommendation (MPA) + met daily protein intake recommendation (DPR), (2) MPA + did not meet daily protein intake recommendation (nDPR), (3) did not meet physical activity (nMPA) + DPR, (4) nMPA + nDPR. Similarly, physical activity and the three aspects of protein sources were integrated into four groups respectively: (1) MPA + good classification, (2) MPA + fair/poor classification, (3) nMPA + good classification and (4) nMPA + fair/poor classification.

### 2.5. Possible Confounding Variables

The following sample demographics were included in the analyses: age, sex, race/ethnicity (White, Black, Hispanic, Other), education (high school or less, some college or more), and family income to poverty ratio (0–5) [20]. In addition, weight status (underweight, normal, overweight and obese), which was defined by body mass index (BMI, kg/m^2^) based on Centers for Disease Control and Prevention’s weight status cut points [27], and total daily energy intake were included as confounding variables [28,29].

### 2.6. Statistical Analysis

Respondent characteristics at different lean mass levels were examined by using linear regression models for continues variables (e.g., age, BMI, etc.) and logistic regression models for categorical variables (e.g., sex, race/ethnicity), respectively. To examine associations between physical activity, total protein intake, protein sources and lean mass, β [95% confidence intervals (CIs), *p*-values] were obtained by performing multiple linear regression models. To examine the difference in the lean mass levels by the integrated physical activity and protein groups, odds ratios (95%CIs, *p*-values) were obtained by performing multinomial logistic regression models. All multiple models were adjusted for age, race/ethnicity, education, ratio of family income to poverty, weight status, and total daily energy intake. Analyses were separated by sex due to the physiological difference between males and females regarding muscle growth and development throughout adulthood [30]. All analyses were conducted using SAS 9.4 (SAS Institute Inc., Cary, NC, USA) and the 6-year combined sample weight which was constructed using the dietary two-day sample weights according to NHANES’s Analytic Guidelines [31] with statistical significance level was set at *p* < 0.05.

## 3. Results

Approximately half (49.6%) of respondents were female, 38.2% were racial/ethnic minorities, 33.6% had high school degree or less, and 16.2% had family income below the poverty line. There were differences in LMI and ALMI levels by sample characteristics. More specifically, white respondents, those who had some college or more, family income at or above poverty, and respondents who were obese had higher LMI and/or ALMI. All models were adjusted for these variables (Table 1).

### 3.1. Total Protein Intake and Lean Mass

**Figure 1:**
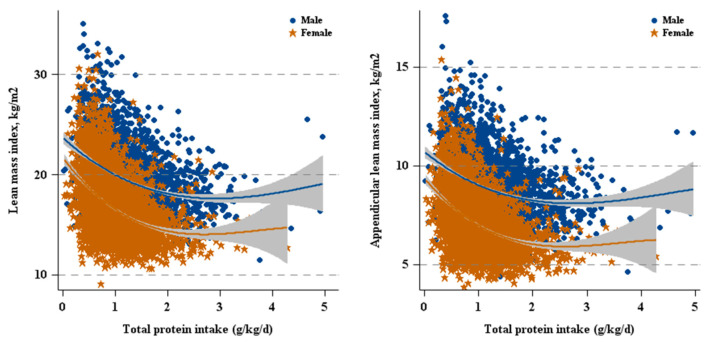
Total protein intake versus lean mass by sex.

As shown in Figure 1 and Table 2, there was not a significant association between total protein intake and lean mass in males. However, daily protein intake was inversely related to LMI (β =−0.84, 95% CI: −1.06–−0.62) and ALMI (β =−0.35, 95% CI: −0.48–−0.22) in females.

### 3.2. Protein Sources and Lean Mass

**Figure 2:**
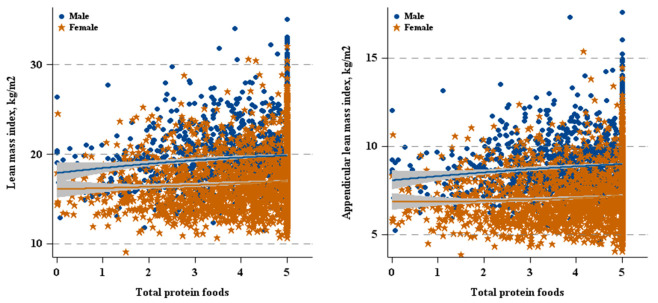
Total protein foods versus lean mass by sex.

**Figure 3:**
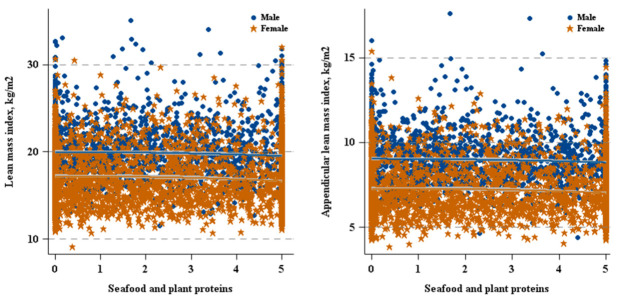
Seafood and plant proteins versus lean mass by sex.

As shown in Figure 2 and Table 2, there was a positively relationship between Total Protein Foods and ALMI in males (β = 0.06, 95% CI: 0.01–0.12). In females, as shown in Figure 2 and Figure 3, and Table 2, both Total Protein Foods (β = 0.04, 95% CI: 0.01–0.08) and Seafood and Plant Proteins (β = 0.02, 95% CI: 0.00–0.04) were positively associated with ALMI.

### 3.3. Physical Activity and Lean Mass

**Figure 4:**
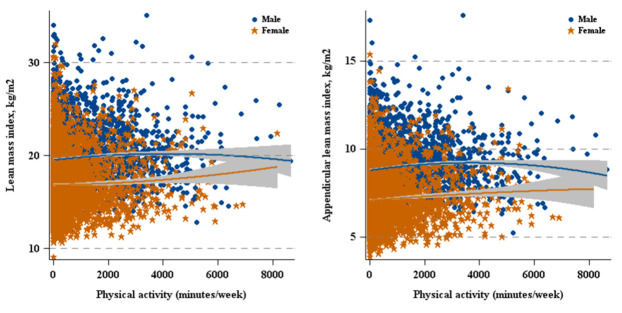
Physical activity versus lean mass by sex.

Significant associations between physical activity and lean mass were identified for both sexes (see Figure 4 and Table 2). In males, every 30 min/week increase in physical activity was associated with an 0.002 kg/m^2^ increase in LMI (β = 0.002, 95% CI: 0.001–0.004) and a 0.001 kg/m^2^ increase in ALMI (β = 0.001, 95% CI: 0.001–0.002). For females, with every 30 min/week increase in physical activity, LMI and ALMI increased by 0.005 kg/m^2^ (β = 0.005, 95% CI: 0.003–0.007) and 0.003 kg/m^2^ (β = 0.003, 95% CI: 0.001–0.004) respectively.

### 3.4. Physical Activity, Total Protein Intake, Protein Quality, and Lean Mass

Four different physical activity and protein intake integrated groups were compared with higher and lower lean mass levels (Table 3, Table 4, Table 5 and Table 6). The odds of having higher lean mass levels were lower for males and females in the following groups compared to the referent group MPA + good protein intake (DPR or protein sources classified as good): (1) nMPA + good protein intake (DPR or protein sources classified as good), and/or (2) nMPA + not good protein intake (nDPR or protein sources classified as poor/fair). However, no difference was observed in MPA + not good protein intake (nDPR or protein sources classified as poor/fair), with the exception for total protein intake (Table 3) and Total Protein Foods (Table 5) in females.

## 4. Discussion

The present study is the first, to our knowledge, to examine the relationships between total protein intake, sources of protein, physical activity, and lean mass in a representative sample of US adults. Study findings indicate that total protein intake was negatively associated with LMI and/or ALMI but higher quality protein sources (Total Protein Foods, Seafood and Plant Proteins) and physical activity were positively correlated with these indices in males and/or females. Furthermore, respondents who did not meet the physical activity recommendation were more likely to have low LMI and/or ALMI, but differences by sex were identified.

### 4.1. Total Protein Intake and Lean Mass

The present study found that total protein intake was inversely associated with LMI and ALMI in females. However, this association was not observed in males. Existing large-scale studies on total protein intake and DXA-based measures of lean mass are lacking and results of existing studies are inconsistent. Some of the studies that are similar to the present study, found that higher protein intake was associated with lower LMI in postmenopausal women [10] or that total protein intake is not related to lean mass in older adults [32]; Other studies have found that higher protein intake is associated with higher leg lean mass among Massachusetts adults aged 29–86 year [9] or greater lean mass in Taiwanese older adults [33] or better lean mass retention in older adults from two states in the eastern part of the US [34], or Tasmanian older adult from Australia [35]. It is important to note that most of these studies focused on older adults, and none of them examined the relationship between total protein intake and lean mass in a representative sample of US adults. One possible explanation for our study findings might be that despite the general understanding that protein consumption results in an anabolic cascade in both sexes, increasing daily protein consumption beyond the recommended daily intake determined by one’s specific needs (e.g., physical activity level) is not beneficial for males and may even be detrimental to developing and preserving lean mass in females [36,37]. However, the present study cannot prove causality due to the cross-sectional study design. Further research is needed on total protein intake and lean mass in representative adult populations.

### 4.2. Sources of Protein and Lean Mass

A novelty of the present study was to examine the associations between protein sources and lean mass. Since the 2015–2020 Dietary Guidelines highlight consuming a plant-based dietary pattern for better health [19], it is important to evaluate the relationship between dietary protein sources and lean mass using a measure that is not solely based on intake of animal protein. Therefore, the present study examined three main protein sources (Dairy, Total Protein Foods, Seafood and Plant Proteins), and determined that Total Protein Foods consumption was positively associated with ALMI in both males and females, Seafood and Plant Protein Foods consumption only have a positive relationship with ALMI in females. It is possible that consuming Total Protein Foods from various plant and animal sources are beneficial for healthier lean mass. However, it is also possible that certain dietary constituents such as meat, poultry, eggs, which differentiated Total Protein Foods from Seafood and Plant Proteins, might be more influential on lean mass in males [38,39,40,41] since only Total Protein Foods but not Seafood and Plant Proteins had a significant relationship with ALMI in males. It is however beyond the scope of the present cross-sectional study and the specificity of those dietary constituents was not the focus of this study. Furthermore, prior research examining the association between dairy and lean mass has suggested that greater dairy intake promotes lean mass development and preservation [42,43]. Although it is impossible to compare since prior studies only focused on the total protein consumption rather than protein sources, the non-significant results observed in the present study might be due to insufficient variability in dairy. Further longitudinal research to investigating how changes in sources of protein affect lean mass change is warranted.

### 4.3. Physical Activity and Lean Mass

Our finding that physical activity was positively associated with lean mass is supported by prior research [8,11], although previous studies used different analytical approaches (overall vs. sex-specific analysis), outcome measures (leg lean mass vs. lean mass) [8] and samples (British older adults vs. US adults) [11]. As the relationship between physical activity and lean mass by sex has not been examined in a representative sample of US adults, the present study begins to address this knowledge gap. We identified a positive relationship between physical activity and lean mass for males and females, and determined that increasing physical activity was associated with an increased the likelihood of having higher LMI and ALMI for men and women. A possible explanation for this finding is that when comparing across a diverse population, being physically active is beneficial for lean mass regardless of the types and intensity levels of the activity performed [44].

### 4.4. Physical Activity, Total Protein Intake, Sources of Protein, and Lean Mass

A novel aspect of the present study was to explore the integrated physical activity and protein intake differences by lean mass levels. Our findings showed that males, regardless of protein intake, who did not meet the physical activity recommendation were more likely to have low lean mass than (1) males who met both physical activity and daily protein intake recommendations or (2) males who met the physical activity recommendation and consumed higher quality protein sources. However, there was no statistically significant difference in the lean mass levels among males who met the physical activity recommendation and did not meet daily protein intake guideline or males who met the physical activity recommendation and consumed protein sources that were classified as poor/fair. A similar pattern was observed in females; however, females who met the physical activity recommendation and did not comply with daily protein intake guideline were more likely to have high ALMI than those who met the physical activity recommendation and complied with daily protein intake guideline. The possible explanation for these variations may be due to overall differences in dietary patterns between males and females [18]. Nevertheless, the results highlight the importance of physical activity for lean mass. However, the benefit of adhering to the recommended daily protein intake allowance or protein aspect of 2015–2020 Dietary Guidelines for Americans should not be underestimated either. Further research is warranted to investigate the effects of physical activity together with dietary protein on lean mass overtime.

### 4.5. Study Strengths and Limitations

This is the first study examining sex-specific relationships between physical activity, total protein intake, sources of protein, and lean mass in a representative sample of US adults. A strength of the study is that sources of protein were assessed in the context of overall dietary pattern and measured using HEI-2015 which reflects 2015–2020 Dietary Guidelines for Americans. An additional study strength is that lean mass was measured by DXA which is the most accurate measure of lean mass. The DXA instrument is limited by its size and weight of people that can be measured [20] which may limit the generalizability. Study limitations include the use of self-reported measures to assess physical activity and dietary protein. Also, the Global Physical Activity Questionnaire does not differentiate between the type of exercise that respondents did even though type of exercise such as resistance training are more beneficial to lean mass [45]. However, the Global Physical Activity Questionnaire and 24-h dietary recalls both are widely used and validated instruments [20,24]. Lastly, this study is limited by its cross-sectional design and not being able to determine the directionality of the relationships.

## 5. Conclusions

The present study found that higher total protein intake was related to lower lean mass in females only; bettering Total Protein Foods and Seafood and Plant Proteins consumptions were associated with higher lean mass in females and/or males; increased physical activity was associated with higher lean mass regardless of sex. Furthermore, males and females who did not meet physical activity recommendation were more likely to have low lean mass and there were variations by sex. Given the adverse association between excess protein intake and lean mass in females, careful consideration is needed for determining optimal sex-specific protein recommendations as well as the dietary sources of those proteins. In conclusion, these findings may have important health implication for lean mass improvement and retention in adults. Further studies are warranted to investigate the influence of changes in physical activity, total protein intake, and sources of protein on lean mass.

## Figures and Tables

**Table 1:** Subjects characteristics by lean mass levels, NHANES 2011–2016.

Variables	Total	Lean Mass Index (kg/m^2^)				Appendicular Lean Mass Index (kg/m^2^)			
		Low (1st Tertile)	Middle (2nd Tertile)	High (3rd Tertile)	*p*-Value	Low (1st Tertile)	Middle (2nd Tertile)	High (3rd Tertile)	*p*-Value
	*n* = 7547	*n* = 2825 (38.4%)	*n* = 2360 (31.6%)	*n* = 2362 (30.0%)		*n* = 2865 (39.4%)	*n* = 2345 (31.2%)	*n* = 2337 (29.4%)	
Female, *n*%	3837 (49.6)	2132 (76.5)	999 (38.6)	706 (26.6)	<0.001 *	2281 (80.5)	1016 (38.7)	540 (19.8)	<0.001 *
Age, yrs	39.7 ± 0.3	39.5 ± 0.5	40.0 ± 0.4	39.8 ± 0.4	0.695	40.2 ± 0.5	40.2 ± 0.5	38.5 ± 0.4	0.005 *
Race/ethnicity, *n*%									
White	2794 (61.8)	1110 (65.5)	850 (62.2)	834 (56.8)	<0.001 *	1165 (66.2)	865 (62.7)	764 (55.2)	<0.001 *
Black	1692 (11.7)	490 (8.6)	535 (12.1)	667 (15.4)	<0.001 *	433 (7.4)	492 (11.1)	767 (18.1)	<0.001 *
Hispanic	1827 (17.1)	607 (14.4)	563 (16.4)	657 (21.5)	<0.001 *	672 (15.0)	585 (17.1)	570 (19.9)	<0.001 *
Other	1234 (9.3)	618 (11.6)	412 (9.4)	204 (6.3)	<0.001 *	595 (11.4)	403 (9.1)	236 (6.8)	<0.001 *
Education, *n*%									
High school or less	2925 (33.6)	951 (29.4)	949 (33.3)	1025 (39.4)	<0.001 *	1014 (30.1)	948 (34.9)	963 (37.0)	<0.001 *
Some college or more	4621 (66.4)	1874 (70.6)	1410 (66.7)	1337 (60.6)	<0.001 *	1851 (69.9)	1396 (65.1)	1374 (63.0)	<0.001 *
Ratio of family income to poverty, *n*%									
Below poverty (<1.0)	1599 (16.2)	609 (16.0)	502 (16.5)	488 (16.2)	0.934	630 (16.5)	494 (16.4)	475 (15.7)	0.831
At or above poverty (≥ 1.0)	5434 (83.8)	2021 (84.0)	1699 (83.5)	1714 (83.8)	0.934	2042 (83.5)	1689 (83.6)	1703 (84.3)	0.831
Weight, kg	83.4 ± 0.5	70.5 ± 0.6	80.8 ± 0.3	102.8 ± 0.6	<0.001 *	71.7 ± 0.6	81.6 ± 0.4	101.0 ± 0.6	<0.001 *
Height, cm	169.5 ± 0.2	166.7 ± 0.3	170.5 ± 0.3	171.9 ± 0.3	<0.001 *	165.8 ± 0.3	170.4 ± 0.4	173.3 ± 0.3	<0.001 *
Body mass index, kg/m^2^	29.0 ± 0.2	25.2 ± 0.2	27.9 ± 0.1	34.9 ± 0.2	<0.001 *	25.9 ± 0.2	28.3 ± 0.1	33.8 ± 0.2	<0.001 *
Weight status, *n*%									
Underweight (<18.5 kg/m^2^)	124 (1.4)	124 (3.6)	0 (0.0)	0 (0.0)	0.934	123 (3.5)	1 (0.0)	0 (0.0)	0.831
Normal weight (18.5–24.9 kg/m^2^)	2125 (28.7)	1526 (53.7)	586 (25.3)	13 (0.4)	<0.001 *	1367 (47.6)	662 (28.5)	96 (3.8)	<0.001 *
Overweight (25.0–29.9 kg/m^2^)	2343 (32.7)	812 (29.4)	985 (45.4)	546 (23.4)	<0.001 *	853 (31.1)	828 (37.8)	662 (29.3)	0.001 *
Obese (≥ 30.0 kg/m^2^)	2911 (36.5)	339 (12.3)	770 (28.4)	1802 (76.2)	<0.001 *	498 (16.8)	837 (32.9)	1576 (66.8)	<0.001 *
Total daily energy intake, kcal	2236.6 ± 14.1	2023.6 ± 29.2	2246.7 ± 23.9	2498.8 ± 29.8	<0.001 *	1989.2 ± 27.0	2265.0 ± 21.2	2537.1 ± 33.7	<0.001 *
Lean mass index, kg/m^2^	18.2 ± 0.1	14.7 ± 0.0	18.1 ± 0.0	22.0 ± 0.1	<0.001 *	15.0 ± 0.1	18.1 ± 0.0	21.8 ± 0.1	<0.001 *
Appendicular lean mass index, kg/m^2^	7.9 ± 0.0	6.2 ± 0.0	8.0 ± 0.0	9.8 ± 0.0	<0.001 *	6.2 ± 0.0	7.9 ± 0.0	9.9 ± 0.0	<0.001 *
Total protein intake, g/kg/d	1.1 ± 0.0	1.1 ± 0.0	1.1 ± 0.0	1.0 ± 0.0	<0.001 *	1.1 ± 0.0	1.1 ± 0.0	1.0 ± 0.0	<0.001 *
DPR, *n*%	5075 (68.7)	2111 (74.4)	1619 (72.1)	1345 (58.0)	<0.001 *	2057 (71.8)	1612 (71.3)	1406 (61.8)	<0.001 *
HEI-2015 total	53.3 ± 0.4	55.1 ± 0.5	53.2 ± 0.4	51.2 ± 0.5	<0.001 *	54.8 ± 0.6	52.9 ± 0.5	51.6 ± 0.5	<0.001 *
Dairy	5.4 ± 0.1	5.4 ± 0.1	5.5 ± 0.1	5.3 ± 0.1	0.397	5.4 ± 0.1	5.4 ± 0.1	5.3 ± 0.1	0.737
GD, *n*%	2439 (36.5)	939 (35.8)	784 (38.6)	716 (35.0)	0.225	966 (36.3)	766 (37.6)	707 (35.4)	0.568
Total protein foods	4.5 ± 0.0	4.4 ± 0.0	4.5 ± 0.0	4.6 ± 0.0	<0.001 *	4.4 ± 0.0	4.5 ± 0.0	4.6 ± 0.0	<0.001 *
GTP, *n*%	5137 (66.5)	1826 (62.5)	1609 (67.2)	1702 (71.0)	<0.001 *	1853 (62.6)	1590 (66.7)	1694 (71.6)	<0.001 *
Seafood and plant proteins	2.9 ± 0.0	3.0 ± 0.1	3.0 ± 0.1	2.7 ± 0.1	0.003 *	3.0 ± 0.1	2.9 ± 0.1	2.8 ± 0.1	0.125
GSPP, *n*%	3145 (40.9)	1250 (41.7)	1055 (44.5)	840 (36.0)	<0.001 *	1266 (41.9)	1009 (41.5)	870 (39.0)	0.355
Physical activity, min/week	752.4 ± 21.3	636.5 ± 24.1	783.4 ± 36.1	868.2 ± 34.6	<0.001 *	629.8 ± 25.4	739.0 ± 38.9	930.4 ± 35.9	<0.001 *
MPA, *n*%	5086 (69.9)	1791 (67.4)	1645 (72.4)	1650 (70.3)	0.034 *	1782 (66.4)	1592 (69.9)	1712 (74.4)	<0.001 *

Note: Data are presented as weighted mean ± standard errors for continues variables and count (weighted%) for categorical variables, overall *p*-value indicated that whether two or more lean mass levels differed from one another. yrs, years, HEI: Healthy Eating Index, DPR: met daily protein intake recommendation of 0.8 g/kg/d, GD: good dairy (3rd tertile, scored 6.51–10), GTP: total protein foods scored 5 (66%), GSPP: seafood and plant proteins scored 5 (60%), MPA: met recommendation of at least 150 min/week moderate intensity or 75 min/week vigorous intensity physical activity or an equivalent combination of both, * *p* < 0.05.

**Table 2:** Relationships of total protein intake, sources of protein, physical activity, and lean mass, NHANES 2011–2016.

Variables	Male		Female	
	Lean Mass Index (kg/m^2^)	Appendicular Lean Mass Index (kg/m^2^)	Lean Mass Index (kg/m^2^)	Appendicular Lean Mass Index (kg/m^2^)
Total protein intake – per increase of 1 g/kg/d	−0.23 (−0.50–0.03, 0.086)	−0.01 (−0.15–0.13, 0.887)	−0.84 (−1.06–−0.62, <0.001 *)	−0.35 (−0.48–−0.22, <0.001 *)
Dairy—per increase of 1 point	0.01 (−0.02–0.03, 0.476)	0.01 (−0.01–0.02, 0.321)	0.01 (−0.02–0.04, 0.626)	−0.00 (−0.02–0.02, 0.982)
Total protein foods—per increase of 1 point	0.10 (−0.01–0.20, 0.071)	0.06 (0.01–0.12, 0.034 *)	0.05 (−0.02–0.12, 0.152)	0.04 (0.01–0.08, 0.028 *)
Seafood and plant proteins—per increase of 1 point	0.02 (−0.01–0.06, 0.191)	0.02 (−0.00–0.04, 0.118)	0.01 (−0.03–0.05, 0.545)	0.02 (0.00–0.04, 0.016 *)
Physical activity – per increase of 30 min/week	0.002 (0.001–0.004, 0.017 *)	0.001 (0.001–0.002, 0.008 *)	0.005 (0.003–0.007, <0.001 *)	0.003 (0.001–0.004, <0.001 *)

Note: β (95% confidence interval, *p*-value) obtained by performing multiple linear regression models, and adjusted for age, race/ethnicity, education level, ratio of family income to poverty, weight status, total daily energy intake, * *p* < 0.05.

**Table 3:** The integrated physical activity and total protein intake differences by lean mass levels, NHANES 2011–2016.

Variables	MPA + DPR	MPA + nDPR	nMPA + DPR	nMPA + nDPR
	OR (95%CI, *p*-value) ^@^			
**Male**				
Lean mass index (kg/m^2^)				
2nd tertile vs. 1st tertile	REF	1.10 (0.57–2.13, 0.779)	0.83 (0.56–1.23, 0.348)	0.61 (0.29–1.26, 0.174)
3rd tertile vs. 1st tertile	REF	0.82 (0.43–1.57, 0.538)	0.58 (0.37–0.91, 0.019*)	0.67 (0.35–1.28, 0.218)
Appendicular lean mass index (kg/m^2^)				
2nd tertile vs. 1st tertile	REF	0.99 (0.53–1.82, 0.962)	1.03 (0.67–1.57, 0.907)	0.43 (0.20–0.91, 0.029 *)
3rd tertile vs. 1st tertile	REF	0.70 (0.40–1.22, 0.203)	0.52 (0.34–0.80, 0.004 *)	0.44 (0.26–0.76, 0.004 *)
**Female**				
Lean mass index (kg/m^2^)				
2nd tertile vs. 1st tertile	REF	0.65 (0.41–1.05, 0.075)	0.45 (0.29–0.70, <0.001 *)	0.36 (0.24–0.54, <0.001 *)
3rd tertile vs. 1st tertile	REF	2.15 (0.99–4.71, 0.054)	0.76 (0.41–1.41, 0.372)	1.12 (0.59–2.11, 0.733)
Appendicular lean mass index (kg/m^2^)				
2nd tertile vs. 1st tertile	REF	0.69 (0.41–1.15, 0.146)	0.66 (0.42–1.02, 0.061)	0.46 (0.31–0.69, <0.001 *)
3rd tertile vs. 1st tertile	REF	2.10 (1.01–4.36, 0.047 *)	0.72 (0.38–1.36, 0.308)	1.32 (0.79–2.20, 0.276)

Note: ^@^ obtained by performing multinomial logistic regression models and adjusted for age, race/ethnicity, education level, ratio of family income to poverty, weight status, and total daily energy intake, OR: odds ratio, CI: confidence interval, REF: reference group, MPA: met physical activity recommendation of at least 150 min/week moderate intensity or 75 min/week vigorous intensity physical activity or an equivalent combination of both, nMPA: did not meet physical activity recommendation, DPR: met dietary protein recommendation of 0.8 g/kg/d, nDPR: did not meet the dietary protein intake recommendation, * *p* < 0.05.

**Table 4:** The integrated physical activity and dairy differences by lean mass levels, NHANES 2011–2016.

Variables	MPA + GD	MPA + PFD	nMPA + GD	nMPA + PFD
	OR (95%CI, *p*-value) ^@^			
**Male**				
Lean mass index (kg/m^2^)				
2nd tertile vs. 1st tertile	REF	0.95 (0.65–1.39, 0.783)	0.96 (0.46–2.00, 0.914)	0.67 (0.43–1.05, 0.079)
3rd tertile vs. 1st tertile	REF	0.90 (0.54–1.51, 0.69)	0.73 (0.44–1.21, 0.214)	0.57 (0.35–0.94, 0.028 *)
Appendicular lean mass index (kg/m^2^)				
2nd tertile vs. 1st tertile	REF	0.97 (0.63–1.49, 0.892)	0.61 (0.30–1.23, 0.163)	0.97 (0.58–1.62, 0.903)
3rd tertile vs. 1st tertile	REF	0.84 (0.55–1.30, 0.429)	0.48 (0.29–0.77, 0.003 *)	0.52 (0.35–0.77, 0.002 *)
**Female**				
Lean mass index (kg/m^2^)				
2nd tertile vs. 1st tertile	REF	0.98 (0.59–1.64, 0.941)	0.53 (0.31–0.91, 0.023 *)	0.46 (0.29–0.75, 0.002 *)
3rd tertile vs. 1st tertile	REF	1.18 (0.68–2.03, 0.552)	0.67 (0.37–1.20, 0.173)	0.60 (0.34–1.07, 0.08)
Appendicular lean mass index (kg/m^2^)				
2nd tertile vs. 1st tertile	REF	1.22 (0.77–1.92, 0.388)	0.92 (0.58–1.46, 0.723)	0.67 (0.48–0.94, 0.021 *)
3rd tertile vs. 1st tertile	REF	1.38 (0.78–2.44, 0.256)	0.75 (0.38–1.52, 0.422)	0.81 (0.49–1.33, 0.398)

Note: ^@^ obtained by performing multinomial logistic regression models and adjusted for age, race/ethnicity, education level, ratio of family income to poverty, weight status, and total daily energy intake, OR: odds ratio, CI: confidence interval, REF: reference group, MPA: met physical activity recommendation of at least 150 min/week moderate intensity or 75 min/week vigorous physical activity or an equivalent combination of both, nMPA: did not meet physical activity recommendation, GD: good dairy (3rd tertile), PFD: 1st and 2nd tertiles of dairy distribution. * *p* < 0.05.

**Table 5:** The integrated physical activity and total protein foods differences by lean mass levels, NHANES 2011–2016.

Variables	MPA + GTP	MPA + PTP	nMPA + GTP	nMPA + PTP
	OR (95%CI, *p*-value) ^@^			
**Male**				
Lean mass index (kg/m^2^)				
2nd tertile vs. 1st tertile	REF	0.93 (0.57–1.49, 0.745)	0.74 (0.50–1.10, 0.137)	0.79 (0.45–1.37, 0.389)
3rd tertile vs. 1st tertile	REF	1.13 (0.63–2.04, 0.678)	0.73 (0.46–1.18, 0.193)	0.57 (0.32–0.99, 0.048 *)
Appendicular lean mass index (kg/m^2^)				
2nd tertile vs. 1st tertile	REF	0.95 (0.57–1.59, 0.852)	1.08 (0.72–1.63, 0.707)	0.55 (0.30–1.02, 0.059)
3rd tertile vs. 1st tertile	REF	0.96 (0.56–1.64, 0.882)	0.71 (0.45–1.10, 0.123)	0.38 (0.22–0.64, <0.001 *)
**Female**				
Lean mass index (kg/m^2^)				
2nd tertile vs. 1st tertile	REF	1.09 (0.63–1.89, 0.748)	0.59 (0.38–0.90, 0.017 *)	0.38 (0.24–0.62, <0.001 *)
3rd tertile vs. 1st tertile	REF	0.65 (0.36–1.16, 0.14)	0.57 (0.34–0.94, 0.028 *)	0.37 (0.23–0.61, <0.001 *)
Appendicular lean mass index (kg/m^2^)				
2nd tertile vs. 1st tertile	REF	1.11 (0.69–1.80, 0.66)	0.76 (0.51–1.14, 0.182)	0.58 (0.38–0.87, 0.01 *)
3rd tertile vs. 1st tertile	REF	0.58 (0.35–0.98, 0.043 *)	0.57 (0.35–0.92, 0.022 *)	0.50 (0.26–0.96, 0.039 *)

Note: ^@^ obtained by performing multinomial logistic regression models and adjusted for age, race/ethnicity, education level, ratio of family income to poverty, weight status, and total daily energy intake, OR: odds ratio, CI: confidence interval, REF: reference group, MPA: met physical activity recommendation of at least 150 min/week moderate intensity or 75 min/week vigorous intensity physical activity or an equivalent combination both, nMPA: did not meet physical activity recommendation, GTP: total protein foods scored 5 (66%), PTP: total protein foods scored < 5 (34%), * *p* < 0.05.

**Table 6:** The integrated physical activity and seafood and plant proteins differences by lean mass levels, NHANES 2011–2016.

Variables	MPA + GSPP	MPA + PSPP	nMPA + GSPP	nMPA + PSPP
	OR (95%CI, *p*-value) ^@^			
**Male**				
Lean mass index (kg/m^2^)				
2nd tertile vs. 1st tertile	REF	0.74 (0.54–1.02, 0.064)	0.72 (0.42–1.24, 0.231)	0.63 (0.37–1.07, 0.085)
3rd tertile vs. 1st tertile	REF	1.10 (0.70–1.74, 0.678)	0.89 (0.51–1.56, 0.681)	0.61 (0.37–1.00, 0.049 *)
Appendicular lean mass index (kg/m^2^)				
2nd tertile vs. 1st tertile	REF	0.94 (0.65–1.35, 0.719)	1.04 (0.62–1.75, 0.874)	0.72 (0.42–1.23, 0.227)
3rd tertile vs. 1st tertile	REF	0.86 (0.57–1.29, 0.45)	0.69 (0.38–1.27, 0.231)	0.44 (0.28–0.69, <0.001 *)
**Female**				
Lean mass index (kg/m^2^)				
2nd tertile vs. 1st tertile	REF	0.68 (0.40–1.14, 0.140)	0.43 (0.24–0.76, 0.004 *)	0.37 (0.23–0.60, <0.001 *)
3rd tertile vs. 1st tertile	REF	0.82 (0.44–1.50, 0.504)	0.48 (0.25–0.92, 0.028 *)	0.51 (0.28–0.95, 0.033 *)
Appendicular lean mass index (kg/m^2^)				
2nd tertile vs. 1st tertile	REF	0.75 (0.49–1.16, 0.197)	0.62 (0.37–1.02, 0.061)	0.54 (0.33–0.88, 0.014 *)
3rd tertile vs. 1st tertile	REF	0.65 (0.40–1.04, 0.072)	0.60 (0.30–1.21, 0.152)	0.44 (0.24–0.83, 0.012 *)

Note: ^@^ obtained by performing multinomial logistic regression models and adjusted for age, race/ethnicity, education level, ratio of family income to poverty, weight status, and total daily energy intake, OR: odds ratio, CI: confidence interval, REF: reference group, MPA: met physical activity recommendation of at least 150 min/week moderate intensity or 75 min/week vigorous intensity physical activity or an equivalent combination of both, nMPA: did not meet physical activity recommendation, GSPP: seafood and plant proteins scored 5 (60%), PSPP: seafood and plant proteins scored < 5 (40%), * *p* < 0.05.
